# MicroRNAs regulate neuronal plasticity and are involved in pain mechanisms

**DOI:** 10.3389/fncel.2014.00031

**Published:** 2014-02-11

**Authors:** Sara Elramah, Marc Landry, Alexandre Favereaux

**Affiliations:** ^1^Interdisciplinary Institute for Neuroscience, UMR 5297, University of BordeauxBordeaux, France; ^2^Interdisciplinary Institute for Neuroscience, UMR 5297, Centre National de la Recherche ScientifiqueBordeaux, France

**Keywords:** microRNA, gene expression, neuron, plasticity, pain

## Abstract

MicroRNAs (miRNAs) are emerging as master regulators of gene expression in the nervous system where they contribute not only to brain development but also to neuronal network homeostasis and plasticity. Their function is the result of a cascade of events including miRNA biogenesis, target recognition, and translation inhibition. It has been suggested that miRNAs are major switches of the genome owing to their ability to regulate multiple genes at the same time. This regulation is essential for normal neuronal activity and, when affected, can lead to drastic pathological conditions. As an example, we illustrate how deregulation of miRNAs can affect neuronal plasticity leading to chronic pain. The origin of pain and its dual role as a key physiological function and a debilitating disease has been highly debated until now. The incidence of chronic pain is estimated to be 20–25% worldwide, thus making it a public health problem. Chronic pain can be considered as a form of maladaptive plasticity. Long-lasting modifications develop as a result of global changes in gene expression, and are thus likely to be controlled by miRNAs. Here, we review the literature on miRNAs and their targets responsible for maladaptive plasticity in chronic pain conditions. In addition, we conduct a retrospective analysis of miRNA expression data published for different pain models, taking into account recent progress in our understanding of the role of miRNAs in neuronal plasticity.

## INTRODUCTION

MicroRNAs (miRNAs) are non-coding, endogenous ~23 nucleotide (nt) RNAs that regulate target-gene expression by either translation inhibition or mRNA degradation (Bartel, 2009). They were first discovered in *C. elegans* where *lin-4* plays a critical role in the developmental orchestration (Lee et al., 1993). Since then, the number of identified miRNAs has reached a thousand in many species. Owing to their uncommon targeting properties, a single miRNA species can bind and regulate multiple targets, and it is now thought that the expression of most, if not all, genes involves modulation by miRNAs. Thus, the “macro switch” function of miRNAs in neuronal development is now well documented (Li and Jin, 2010). More recently, accruing evidence established miRNAs as essential regulators of proper neuronal function. In particular, it was demonstrated that miRNAs regulate many proteins involved in neuronal adaptation to network activity. miRNAs take part in the morphological and functional changes sustaining neuronal plasticity. For instance, miR-134 is expressed in hippocampal neurons where it regulates the LIM-domain kinase 1, which represses cofilin, an actin depolymerization factor, thus modifying spine morphology (Schratt et al., 2006). Another example is miR-284, which regulates the expression of the glutamate receptor GluA2 at the neuromuscular junction in *Drosophila* (Karr et al., 2009).

As an actor in neuronal plasticity, miRNAs are likely to play a role in the onset and maintenance of neurological diseases. Indeed screening strategies have identified altered expression of specific miRNAs in Alzheimer’s, Parkinson’s, Huntington’s disease, and Tourette syndrome patients (Johnson et al., 2012; Salta and De Strooper, 2012). While these results raise hope for new treatments, more work is needed to fully differentiate the miRNA changes that trigger pathological mechanisms from those resulting from the disease. A tempting hypothesis is that long-term alteration of miRNA pathways normally regulating basal neuron function could lead to disease. Long-lasting modifications of gene expression are thought to result in chronic disease such as neuropathic pain. Therefore, miRNA deregulation could play a key role in chronic pain.

Here, we briefly examine current knowledge on miRNA biogenesis, target recognition, and regulation. Then, we review the literature to highlight miRNAs that are involved in neuronal plasticity, with a special focus on chronic pain pathologies.

## miRNA BIOGENESIS

To date, miRbase, a specialized miRNA database, comprises 1872 entries for the human genome (Griffiths-Jones et al., 2006; Kozomara and Griffiths-Jones, 2011). miRNA genes are present on all chromosomes, with the exception of the human Y chromosome. Nearly 50% of miRNA-coding genes are situated within the intergenic space and possess their own regulatory elements (Lagos-Quintana et al., 2001; Corcoran et al., 2009). In contrast, 40% of miRNAs genes are positioned within introns (Rodriguez et al., 2004; Smalheiser et al., 2008), and 10% are located within exon terminals. As a consequence, the expression of half of the miRNA genes depends on the regulation of their host gene, so they may be involved in the control of genetic networks related to the expected function of the host gene product (O’Carroll and Schaefer, 2013). An interesting feature is that many miRNA genes are grouped within clusters, with an intergenic distance ranging from 0.1 to 50 kb, and exhibit a similar expression pattern (Baskerville and Bartel, 2005). In addition, miRNAs within clusters are often, but not always, related to each other, while miRNAs from the same family are occasionally clustered (Lagos-Quintana et al., 2001; Lau et al., 2001).

Transcription of miRNAs is performed by either RNA Polymerase II or RNA Polymerase III, producing primary miRNA (pri-miRNA), a large stem-loop structure (3–4 kb in length) with a 5′ cap and a poly(A) tail (Cai et al., 2004; Lee et al., 2004). Then, this pri-miRNA structure is recognized and processed by the microprocessor complex which is composed of the ribonuclease Drosha (RNase III enzyme), the RNA-binding protein (RBP) DGCR8 (DiGeorge syndrome critical region gene 8; Lee et al., 2003; Denli et al., 2004; Gregory et al., 2004) and other auxiliary factors (Han et al., 2004), generating ~60- to 70-nt-long stem-loop precursor miRNAs (pre-miRNA; Morlando et al., 2008). Processing of pri-miRNAs into pre-miRNAs occurs concomitantly with transcriptional events, thus rapidly constituting a nuclear pool of pre-miRNAs (Morlando et al., 2008). The next step to functional miRNAs is the export of pre-miRNAs from the nucleus to the cytoplasm by the karyopherin protein family member, Exportin-5, in a GTP-dependent manner (Yi et al., 2003; Bohnsack et al., 2004; Lund et al., 2004). Once in the cytoplasm, the pre-miRNA is loaded into the RNA-induced silencing complex (RISC) loading complex, where it is further processed into a ~21-nt-long miRNA duplex by Dicer, a type-III ribonuclease (Bernstein et al., 2001; Hutvágner et al., 2001; Ketting et al., 2001; Knight and Bass, 2001). At this stage, only one strand is finally incorporated into the RISC, the “guide” strand, whereas the other strand called “the passenger” is likely degraded. The criteria defining which of the two strands is loaded into the RISC still need to be clarified but it appears that, in most of the cases, it is the one whose 5′ end is less tightly paired (Khvorova et al., 2003; Schwarz et al., 2003).

Another processing pathway independent of the Drosha/DGCR8 microprocessor machinery and previously discovered in *Drosophila* and *C. elegans* (Okamura et al., 2007; Ruby et al., 2007a) has been recently identified in mammals. The so-called mirtons are directly spliced from the introns of mRNA coding genes, directly exported to the cytoplasm and processed by Dicer (Okamura et al., 2007; Ruby et al., 2007a).

## TARGET RECOGNITION AND TRANSLATION INHIBITION

### BASICS OF miRNA:mRNA INTERACTIONS

Loading of the “guide” strand into the Argonaute protein of the RISC makes it functional and ready to generate mRNA inhibition (Hutvágner and Zamore, 2002; Mourelatos et al., 2002). The mechanisms and the consequent magnitude of this regulation are dependent on the characteristics of the miRNA:target mRNA binding. Thus, in metazoans, extensive base pairing has been demonstrated to induce mRNA cleavage (Hutvágner and Zamore, 2002; Rhoades et al., 2002; Yekta et al., 2004), whereas imperfect binding leads to target repression through translational inhibition and/or mRNA destabilization (Lee et al., 1993; Wightman et al., 1993; Lim et al., 2005). In addition, the location of miRNA binding sites in the target mRNA plays a predominant role on the efficacy of the regulation. Although binding of miRNA has been demonstrated in the 5′ untranslated region (UTR) and the open reading frame (ORF) of mRNA (Easow et al., 2007; Lytle et al., 2007; Chi et al., 2009; Leung et al., 2011), these sites are less effective than those located in the 3′UTR (Kloosterman et al., 2004; Farh et al., 2005; Lewis et al., 2005; Lim et al., 2005; Grimson et al., 2007; Ruby et al., 2007b; Baek et al., 2008). This might reflect the fact that in 5′UTR and ORF, the translation machinery has a higher affinity than the RISC, thus displacing the silencing complex (Bartel, 2009).

The nucleotide sequence in the target mRNA that is engaged in miRNA binding is called the miRNA recognition element (MRE; Shukla et al., 2011) or “seed region” (Bartel, 2004). miRNA target recognition and seed region classification have been extensively reviewed previously (see Bartel, 2009). The composition of this seed region varies but always involves a sequence with conserved Watson–Crick pairing (i.e., the hydrogen bonding that pairs guanine–cytosine and adenine–thymine) to the 5′ region of the miRNA centered on nucleotides 2–7 (Bartel, 2009). Several types of seed regions exist depending on the length and composition of the sequence involved in miRNA:mRNA binding, resulting in different affinities and inhibition properties. These include canonical sites with a 6- to 8-nt match between miRNA and mRNA, and non-canonical sites that include additional pairing at the 3′ end of the miRNA. Additional factors have been shown to affect miRNA seed efficacy such as AU-rich sites, which have been shown to be more effective (Lewis et al., 2005; Grimson et al., 2007; Nielsen et al., 2007; Pasquinelli, 2012).

Other factors include the position of the seed within the 3′UTR of the target-mRNA (Grimson et al., 2007), with sites at the extremities of the 3′UTR being more accessible than those in the middle because long UTRs may form occlusive interactions, thus greatly reducing miRNA site accessibility.

Another important feature is site multiplicity, where the presence of multiple sites for the same or different miRNAs on the target-mRNA increases the repression response. Multiple sites might contribute to target mRNA inhibition in either a non-cooperative manner or cooperatively. miRNA cooperation results in a stronger repression than the sum of the individual and independent sites. Cooperative regulation implies that seed sequences are within a 40-nt region but with a minimum 8 nt gap between them (Doench et al., 2003; Grimson et al., 2007; Nielsen et al., 2007; Saetrom et al., 2007).

Given the large and growing number of miRNA species, the enormous number of putative target mRNAs and the length of their 3′UTR sequences, the manual prediction of miRNA:mRNA interactions would be impossible. Therefore, computational analysis using algorithms and prediction databases is the usual method to identify miRNA targets. Hence, target prediction programs are based on seed region recognition tools that basically list all the sites with miRNA-binding properties and rank them accordingly to the kind of seed, conservation across evolution and some of the above-mentioned factors known to modulate site efficiency (for review, see Reyes-Herrera and Ficarra, 2012).

However, despite refinements of prediction tools, the percentage of predicted targets that pass the experimental validation stage is still sub-optimal. Therefore, it often constitutes a time-consuming bottleneck in miRNA studies that involve complex experimental paradigms. In line with this, recent data suggest that miRNA:mRNA interactions sometimes involve the 3′ end of miRNAs with little evidence for 5′ contacts, and some of these interactions have been shown to be functional (Helwak et al., 2013). In addition, other parameters not taken into account in these algorithms could affect the accuracy of target prediction tools. For instance, there is the importance of the secondary structure of RNA and its association with RBPs, which influence target site accessibility, and thereby its regulation (Brodersen and Voinnet, 2009).

### MECHANISMS OF miRNA-MEDIATED INHIBITION OF TRANSLATION

miRNA binding to its target mRNA is the starting point of a post-transcriptional regulation of gene expression occurring in the cytoplasm, mostly by translational repression or mRNA degradation (Valencia-Sanchez et al., 2006; Jackson and Standart, 2007; Nilsen, 2007; Pillai et al., 2007; Standart and Jackson, 2007). In fact, microRNA-induced silencing complex (miRISC)-mediated gene inhibition has been demonstrated to arise from three putative mechanisms: (i) site-specific cleavage, (ii) enhanced mRNA decay, and (iii) translational inhibition. Thanks to recent studies combining cutting-edge transcriptomic and proteomic analyses, a genome-wide view of the changes elicited by miRNA down or up-regulation on the protein output is now available (Baek et al., 2008; Selbach et al., 2008; Hendrickson et al., 2009; Guo et al., 2010). These experimental data are critical for validating the prediction tools for miRNA targeting as well as for comparing the respective roles of the different modes of action driven by miRNAs.

#### Site-specific cleavage

This Ago2-mediated process requires full complementarity between miRNA and its target (Hammond et al., 2001; Hutvágner and Zamore, 2002), which seems to be very limited in mammals (Yekta et al., 2004; Hornstein et al., 2005). Loading of the miRNA into the Ago2 protein involves conformational changes which should enhance Watson–Crick pairing to the target mRNA. In contrast to this rare event, translational inhibition or mRNA degradation, which both result from partial binding of miRNA to mRNA, seem to be the most widely occurring mechanisms in mammals (for review, see Fabian et al., 2010).

#### Translational repression

Protein translation repression by miRNAs may result from various mechanisms: inhibition of translation initiation, inhibition of translation elongation; protein degradation while translation is being processed, premature termination of the translation (also known as ribosome drop-off); or a combination of some of these mechanisms (Bagga et al., 2005; Pillai et al., 2005; Nottrott et al., 2006; Petersen et al., 2006).

At the translation initiation step, miRNAs can inhibit protein production by disrupting the interaction between the 5′-cap and the 3′-poly(A) tail of the mRNA. The mechanisms involved are not fully understood but it is thought that Ago could compete with eukaryotic translation initiation factor 4 for mRNA-cap binding (Humphreys et al., 2005; Kiriakidou et al., 2007). Furthermore, miRNAs could prevent translation by inhibiting the association of the ribosomal subunits (Chendrimada et al., 2007; Thermann and Hentze, 2007).

At the post-initiation stage, translation inhibition by miRNAs could be mediated by premature termination and ribosome drop-off (Petersen et al., 2006), stalling of the elongation by slowing down the ribosome process (Rüegsegger et al., 2001; Mootz et al., 2004), or co-translational degradation of nascent polypeptides (Nottrott et al., 2006). However, experimental evidence for these mechanisms and explaining their interdependence is still only partial. As summarized by Filipowicz et al. (2008), the inhibition of translation at the initiation or elongation phase is perhaps not exclusive and it is likely that initiation is always inhibited. However, ribosomes queuing on the mRNA when elongation is also inhibited may hide this obligate mechanism (Filipowicz et al., 2008).

#### Enhanced mRNA decay

Large-scale analyses of the expression of miRNAs and their predicted mRNA targets have shown an inverse correlation, strongly suggesting that mRNA degradation is a hallmark of miRNA-mediated inhibition of transcripts (Chekulaeva and Filipowicz, 2009). A likely scenario relies on the deadenylation of the mRNA target, which would prevent circularization of the transcript. Circularization is an essential mechanism for mRNA translation that also prevents mRNA degradation, since linear transcripts are more prone to degradation (Wakiyama et al., 2007). Nevertheless, whether deadenylation precedes (Wakiyama et al., 2007; Iwasaki et al., 2009) or follows (Fabian et al., 2009; Zdanowicz et al., 2009) translational inhibition is still an unresolved issue.

Beyond the question of the mechanisms responsible for mRNA translation inhibition, there is the issue of the sub-cellular localization of this regulation. The most popular hypothesis posits that mRNAs under miRNA-mediated inhibition are stored (Brengues et al., 2005; Bhattacharyya et al., 2006) and degraded (Eulalio et al., 2007; Parker and Sheth, 2007) into cytoplasmic particles called processing bodies or glycine-tryptophan (GW) bodies. These cytoplasmic foci are free of translational machinery but are enriched in proteins that are required for efficient mRNA inhibition (Jakymiw et al., 2005; Liu et al., 2005; Rehwinkel et al., 2005; Behm-Ansmant et al., 2006).

## miRNAs ARE REGULATORS OF NEURONAL ACTIVITY

Although the way miRNAs regulate their targets is still imperfectly understood, it is widely accepted that they play a major role in gene expression regulation. As such, miRNA have a crucial function in neuronal development where they seem to act as a master switch of the genome (for review, see Sun et al., 2013). In the mature nervous system, increasing evidence suggests that miRNA are important for normal neuronal function (Rajasethupathy et al., 2009; Edbauer et al., 2010; Cohen et al., 2011; Tognini et al., 2011; Dorval et al., 2012; Hsu et al., 2012; Lee et al., 2012; Saba et al., 2012).

The role of miRNA in neuronal function intermingles (i) miRNA that regulates neuronal activity with (ii) neuronal activity that modulates miRNA expression, which could in turn regulate targets with an impact on neuronal activity (feed-forward loop). In addition, neuronal activity should not be considered as a whole but as the output of thousands of synaptic inputs. Upon stimulation, single synapses or sets of synapses can go through intense and selective modifications in an independent manner for long periods of time (Martin and Kosik, 2002; Nelson and Turrigiano, 2008; Wibrand et al., 2010). Although short-term modifications of synapses could mainly be the result of post-translational events, long-term changes require regulation of gene expression at the transcriptional and post-transcriptional levels (Martin and Kosik, 2002; Wibrand et al., 2010). Thus, local post-transcriptional regulation regulated by activity is certainly a key element for the maintenance and plasticity of neural connections (Ashraf and Kunes, 2006; Sutton and Schuman, 2006; Bramham and Wells, 2007). Indeed, it has been demonstrated that specific mRNAs and components of the translational machinery, including ribosomes and other non-coding RNAs, are localized to dendritic regions of neurons, where they are likely to serve local translation (Steward and Levy, 1982; Davis et al., 1987; Torre and Steward, 1992; Trembleau et al., 1994; Landry and Hökfelt, 1998; Steward and Schuman, 2001). Localization of mRNAs into dendrites is an active phenomenon involving the 3′UTR sequence of messenger where RBPs bind to initiate transport (for review, see Doyle and Kiebler, 2011; Goldie and Cairns, 2012). In particular, some mRNAs are located at the post-synaptic density (PSD) as polyribosome structures (Wells, 2012). Some of these mRNAs encode proteins such as kinases and translational control factors, which are attractive candidates to mediate synaptic changes (Eberwine et al., 2001; Steward and Schuman, 2001). Interestingly, the number of polyribosome-containing spines increases in response to the long-term potentialization (LTP) protocol (Ostroff et al., 2002), and their PSD area enlarges when compared to polyribosome-free spines. This indicates that the presence of polyribosomes produces these structural changes, which were previously ascribed to changes in synaptic strength (Martin and Kosik, 2002). It has been suggested that this local translation mechanism could play a major role in synaptic plasticity and therefore contribute to the molecular basis of learning and memory (Kang and Schuman, 1996; Campbell and Holt, 2001; Kim et al., 2004).

**FIGURE 1:**
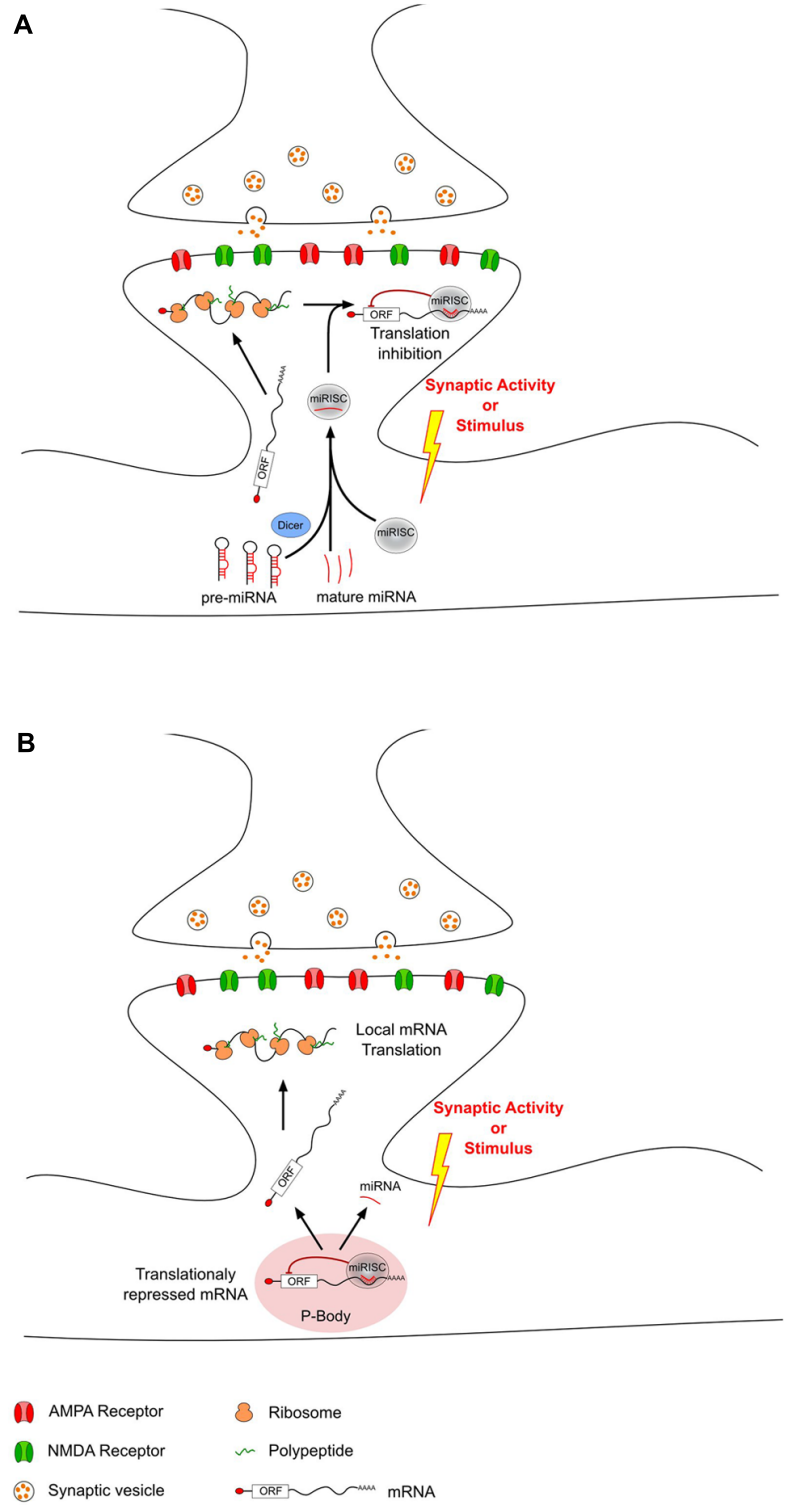
**MicroRNAs can modulate local translation in response to changes in synaptic activity.** After transport from the soma to the dendrites, some mRNAs are locally translated into proteins. This local translation mechanism can be modulated in response to synaptic activity changes by dendritic miRNAs that have also been transported from the soma. This miRNA-driven mechanism can either decrease **(A)** or increase **(B)** local translation. **(A)** Decrease of local translation could be the result of an activity-related miRNA loading into miRISC and specific binding to target mRNAs. **(B)** Increase of translation may be the consequence of an unbinding of miRNA from target-mRNAs which could then escape P-body and undergo local translation.

Recently, miRNAs have been proposed as mediators regulating local protein synthesis at the synaptic level. miRNAs are enriched in the brain (Lagos-Quintana et al., 2002; Krichevsky et al., 2003; Kim et al., 2004; Sempere et al., 2004; Kosik and Krichevsky, 2005), where they have been shown to be differently expressed not only in distinct areas (Landgraf et al., 2007; Bak et al., 2008; Olsen et al., 2009), but also at the synaptic level (Pichardo-Casas et al., 2012). Moreover, studies on synaptoneurosomes revealed the abundance of several components of the miRNAs biogenesis pathway and their silencing complex machinery at PSDs (Lugli et al., 2005; Pichardo-Casas et al., 2012). Thus, dendritic mRNA translation can be modulated by miRNAs which have been selectively transported as mature or pre-miRNAs. In response to synaptic activity, this miRNA-driven regulation can either increase or decrease local mRNA translation through different mechanisms (**Figure 1**). Interestingly, not all miRNAs have the same regulation upon stimulus. However, it is not clear whether the involvement of these different types of regulation depends on the type of synaptic activity or on the type of stimulus.

In addition to their role in physiological conditions, accruing evidence suggests that miRNAs play an important role in the pathological mechanisms of neurodegenerative diseases (reviewed in Johnson et al., 2012; Salta and De Strooper, 2012). Hence, alterations of specific miRNAs have been associated with Alzheimer’s, Parkinson’s, Huntington’s disease, and Tourette syndrome. Furthermore, in other debilitating diseases of the nervous system, miRNA deregulation has been correlated with altered neuronal activity, as in chronic pain, which is characterized by increased excitability within the pain circuitry. One of the first demonstrations of the role of miRNAs in chronic pain came from a study by Zhao et al. (2010) where a conditional knockout of Dicer was targeted to sodium channel Nav1.8-positive nociceptor neurons in the dorsal root ganglia (DRG). This impairment of miRNA function induced a strong attenuation of inflammatory pain, suggesting that miRNAs are necessary to enhance excitability of this subpopulation of neurons in response to inflammation mediators. Here, we review specific miRNAs that have been shown to play a role in the regulation of neuronal activity and highlight their possible association with pain processes (summarized in **Table 1**). In some cases, the causal role of miRNA in pain mechanisms is strongly suggested by targeted-mRNAs identification and functional studies. On the other hand, we conducted a retrospective analysis of miRNA expression data published for different pain models and speculated about their potential implication based on their known targets in normal neuronal activity.

**Table 1:** MicroRNAs involved in plasticity and pain mechanisms.

miRNA	Involvement in plasticity mechanisms			Involvement in pain mechanisms		
	Plasticity protocols	Identified targets	Reference	Pain models	Identified targets	Reference
miR-134	BDNF exposure	LIMK1	Schratt et al. (2006)	Inflammation/CCI	ND	Bai et al. (2007), Genda et al. (2013)
miR-132	Bicuculline/KCl/monocular deprivation	P250GAP /MeCP2	Vo et al. (2005), Klein et al. (2007), Wayman et al. (2008), Mellios et al. (2011), Tognini et al. (2011)	CRPS/CCI	ND	Orlova et al. (2011), Arai et al. (2013)
miR-188	Long-term-potentiation	Nrp-2	Lee et al. (2012)	CCI	ND	Arai et al. (2013)
miR-29a/b	Psychoactive drugs	Arpc3	Lippi et al. (2011)	Inflammation/CRPS/ CCI	ND	Bai et al. (2007), Orlova et al. (2011), Genda et al. (2013)
miR-181a	Psychoactive drugs	GluA2	Schaefer et al. (2010), Chandrasekar and Dreyer (2011), Saba et al. (2012)	Neuropathic/ zymosan-induced pelvic pain	GABA_Aα-1_	Li et al. (2013), Sengupta et al. (2013)
miR-219	Disturbance of NMDA signaling	CaMKIIγ	Kocerha et al. (2009)	Traumatic spinal cord injury/CCI	ND	Liu et al. (2009), Genda et al. (2013)
miR-125	Fragile X syndrome	NR2A	Edbauer et al. (2010)	CCI	ND	Arai et al. (2013)
miR-124	Neuronal differentiation/axon guidance	Sema3A/RhoG/ Zif268	Baudet et al. (2012), Franke et al. (2012), Yang et al. (2012)	Inflammation/nerve injury	MeCP2	Bai et al. (2007), Wu et al. (2011), Willemen et al. (2012), Kynast et al. (2013)

*CCI, chronic constriction injury; CRPS, complex regional pain syndrome; ND, not determined.*

### FUNCTIONAL IMPLICATION OF miRNA IN NEURONAL PLASTICITY AND PAIN SENSITIZATION

One of the first studies reporting the control of local translation by miRNA at the synapse upon synaptic activity showed that miR-134 negatively regulates LIMK1 in an activity-dependent manner (Schratt et al., 2006). LIMK1 is a protein kinase that controls actin filament dynamics through inhibition of actin depolymerizing factor (ADF)/cofilin (Bamburg, 1999). This regulation could be critical for synaptic transmission, synaptic integration, and plasticity since the majority of the excitatory synapses are formed on dendritic spines, which are actin-rich protrusions from the dendritic shaft (Hering and Sheng, 2001; Bonhoeffer and Yuste, 2002; Spruston, 2008). The study demonstrated the compartmentalization of miR-134 and its target LIMK1 mRNA at the synapto-dendritic area. It has been proposed that the miR-134 association with LIMK1 mRNA keeps the LIMK1 mRNA in a dormant state while it is being transported within dendrites to synaptic sites. Over-expression of miR-134 inhibits LIMK1 mRNA local translation, resulting in a negative regulation of the size of the dendritic spines. Exposure of neurons to external stimuli like the neurotrophic factor brain-derived neurotrophic factor (BDNF) reduces miR-134 inhibition on LIMK1 mRNA translation (Schratt et al., 2006). Recently, the same group reported another mechanism by which pre-miR-134 is transported to dendritic sites. Replacing the loop sequence of pre-miR-134 with the loop of a non-dendritic pre-miRNA abolished dendritic accumulation of pre-miR-134. Mutagenesis analysis revealed that the five central loop nucleotides are critical because of an interaction with the Asp-Glu-Ala-His-box (DEAH-box) helicase DEAH box protein 36 (DHX36). DHX36 is known to compete with Dicer *in vitro*, and Argonaute complexes that contain DHX36 are devoid of Dicer activity (Höck et al., 2007). Indeed, knockdown of DHX36 displayed a significant reduction in the percentage of dendritic pre-miR-134, without affecting the global levels of pre-miR-134 or mature miR-134. Consistently with the role of miR-134 on spine morphology, functional experiments revealed that DHX36 negatively regulates dendritic spine morphogenesis in hippocampal neurons (Bicker et al., 2013).

One of the first studies on a possible role of miRNAs in pain showed that miR-134 is modulated in the trigeminal ganglion in response to inflammatory pain (Bai et al., 2007). These authors induced inflammatory pain by injecting complete Freund’s adjuvant (CFA) into the rat masseter muscle, a paradigm known to produce transient inflammation accompanied by allodynia. Interestingly, miR-134 was down-regulated at the onset of pain, 30 min after CFA injection, returned to control level 1 day later, showed an over-expression rebound at day 4 and finally returned to control level at day 12. This phasic deregulation of miR-134 expression suggests that it could be an adaptive response to the early phase of inflammation. Another study in the chronic constriction injury (CCI) model showed that miR-134 was up-regulated in the dorsal horn of the spinal cord at day 14 after surgery (Genda et al., 2013). In this case, miR-134 deregulation occurred later after the onset of the painful behavior and could have been a consequence of the pain mechanisms. In contrast, our own results show that miR-134 was down-regulated 7 days after injury in the dorsal horn of a rat model of sciatic nerve ligation. This was accompanied by an up-regulation of LIMK1, the miR-134 target (A. Salam et al., unpublished data). Therefore, it will be important to undertake functional studies to elucidate the role of miR-134 in chronic pain.

miR-132 is another example of activity-modulated miRNA. Experiments where neuronal activity is increased through a bicuculline-mediated blockade of GABA_A_ inhibitory tone showed a rapid increase in the expression of miR-132 precursor and mature miR-132 (Wayman et al., 2008). Interestingly, this regulation was attenuated by pretreatment with the selective *N*-methyl-D-aspartate receptor (NMDAR) antagonist amino-5-phosphonovaleric acid (APV). In addition, KCl treatment increased transcription of the miR-132 precursor, suggesting that different modalities of neuronal activity stimulation also trigger a similar miR-132 increase. The explanation lies in the pathway leading to miR-132 over-expression. Indeed, inhibitors for CaM kinase, MEK–ERK (mitogen-activated protein kinase kinase–extracellular signal-regulated kinase) or CREB (cAMP response element-binding protein), all prevent miR-132 up-regulation upon activity enhancement, suggesting that miR-132 is regulated predominantly by the CREB pathway. miR-132 directly targets p250GAP, a protein known to inhibit Rho family GTPases (Taniguchi et al., 2003; Zhao et al., 2003). The final outcome of miR-132 over-expression is an increase in dendrite morphogenesis, presumably as a response to Rac activity. Indeed, the same group recently proposed that miR-132 regulates dendritic spinogenesis through the Rac1-Pak actin remodeling pathway (Impey et al., 2010). Another target of miR-132 is methyl CpG-binding protein 2 (MeCP2), which is involved in dendritic development and synaptogenesis and whose modulation can affect synaptic plasticity (Klein et al., 2007). Additional evidence of miR-132 having an impact on neuronal morphology came from transgenic experiments where the genomic locus containing miR-132 (and miR-212 as well) was deleted, leading to a dramatic decrease in dendritic length, arborization, and spine density (Magill et al., 2010). The impact of miR-132 regulation on spinogenesis is revealed in synaptic plasticity paradigms. For instance, *in vitro* short-term plasticity is affected by miR-132 over-expression without altering basal synaptic transmission (Lambert et al., 2010). *In vivo*, short-term recognition memory is impaired by miR-132 over-expression in the perirhinal cortex. This results from a deficit in both long-term depression and potentiation (Scott et al., 2012). In the visual cortex, two recent studies demonstrated that miR-132 is a key component of experience-dependent plasticity. Tognini et al. (2011) showed that monocular deprivation decreased miR-132 expression in the cortex contralateral to the deprived eye. Counterbalancing this miR-132 decrease with miR-132 mimic supplementation completely blocked ocular dominance plasticity. At the same time, Mellios et al. (2011) identified miR-132 as the miRNA whose expression is the most affected by dark rearing and/or monocular deprivation. In addition, miR-132 inhibition occluded ocular dominance plasticity after monocular deprivation, which is associated with an altered maturation of dendritic spines. Finally, a recent study on olfactory bulb neurons born in the neonatal subventricular zone revealed that miR-132 is essential for normal dendritic complexity and spine morphology (Pathania et al., 2012). In conclusion, and as previously proposed (Tognini and Pizzorusso, 2012), finely regulated miR-132 expression seems to be essential for plasticity. Thus, too high an expression would make dendritic spines too stable. Too low an expression would lead to very unstable spines, thus impeding synaptic plasticity.

Vo et al. (2005) demonstrated that BDNF triggered miR-132 up-regulation in cortical neurons. Interestingly, BDNF was recently identified as a modulator of nociception. BDNF is up-regulated in the DRG and the spinal cord in inflammatory and neuropathic pain models (reviewed in Merighi et al., 2008 and Vanelderen et al., 2010, respectively), where it is associated with an increased excitatory synaptic drive. In addition, in most cases, intrathecal BDNF injections exhibit pro-nociceptive effects (Merighi et al., 2008). Thus, one could hypothesize that the effect of BDNF on nociceptive behavior could partially be the result of a BDNF-driven miR-132 over-expression. Thus, BDNF would induce an up-regulation of miR-132, which in turn would increase dendrite morphogenesis and arborization in the nociceptive pathway, resulting in an increased transmission of the pain signal. In patients with complex regional pain syndrome (CRPS), a disabling chronic neuropathic pain affecting one or more extremities, miR-132 blood levels were significantly altered (Orlova et al., 2011). Apart from the direct nociceptive pathway, other nervous structures like the limbic system may influence pain perception. Indeed, the hippocampus is thought to be part of the descending anti-nociceptive system, potentially exerting an inhibitory role in neuropathic pain (Basbaum and Fields, 1979). Arai et al. (2013) screened miRNA expression in the hippocampus of animals submitted to the CCI paradigm of neuropathic pain. miR-132 was down-regulated early after the onset of the neuropathic behavior and stayed under-expressed until the latest time-point analyzed. Thus, it is tempting to hypothesize that this modulation of miR-132 could lower the excitatory drive of the hippocampus and therefore lower the anti-nociceptive effect on the spinal pain pathway.

miR-188 is another activity-regulated miRNA with a proven synaptic plasticity tuning function. Lee et al. (2012) found that miR-188 was up-regulated in hippocampal slices in response to the LTP protocol. Neuropilin-2 (Nrp-2), one of the predicted targets of miR-188, is down-regulated after the LTP procedure and luciferase experiments confirmed the interaction. Nrp-2 is a receptor of semaphorin 3F, which induces the repulsion of neuronal growth cones expressing Nrp-2 (Kolodkin and Ginty, 1997; Kruger et al., 2005). In addition, miR-188 treatment can rescue the decreased miniature excitatory postsynaptic current (mEPSC) frequency and reduction of spine density induced by Nrp-2 over-expression, suggesting that miR-188 plays a role in synaptic plasticity by buffering Nrp-2 expression.

Despite its potentially crucial role in neuronal function, evidence for the involvement of miR-188 in pain mechanisms is very scarce. However, one study reported an altered expression under pain conditions. Arai et al. (2013) quantified a down-regulation of miR-188 in the hippocampus of CCI animals but the significance of this modification remains to be elucidated.

The role of miRNAs in dendritic spine remodeling as a substrate for synaptic plasticity has also been evidenced *in vivo*. Lippi et al. (2011) exposed adult mice to psychoactive drugs like nicotine, cocaine, or amphetamine and quantified miRNA expression in different brain regions. Many miRNAs were regulated in response to these neuroadaptation paradigms, and especially the miR-29a/b locus was consistently over-expressed. *In vitro* experiments confirmed that these miRNAs were up-regulated in an activity-dependent manner. Arpc3 was identified as a target of miR-29a/b. This protein is part of the ARP2/3 actin nucleation complex which plays a critical role in actin branching, a mechanism involved in dendritic spine maturation. Therefore, this work strongly suggests that miR-29a/b regulates the activity-dependent structural plasticity associated with psycho-stimulant exposure.

Several studies identified a deregulation of miR-29a/b in pain conditions. The first evidence came from Bai et al. (2007) who reported a down-regulation of this miRNA in the trigeminal ganglion neurons after inflammatory muscle pain. It was later confirmed in two other pain conditions, the CRPS (Orlova et al., 2011) and the CCI model (Genda et al., 2013). Nevertheless, these studies did not investigate the subsequent regulation of Arpc3 or any other target of miR-29a/b, so the role of miR-29a/b in pain processing still needs to be elucidated.

In the context of chronic exposure to drugs of abuse, Saba et al. (2012) focused their attention on the regulation of miRNAs in the nucleus accumbens. This is one of the most important brain regions in behavioral responses to drugs of abuse and is elicited by dopamine release. Synaptoneurosome preparations revealed a strong enrichment of miR-181a in response to treatment. A previous study showed that miR-181a expression in the striatum was regulated by Ago2 in cocaine addiction (Schaefer et al., 2010). This miRNA was predicted to target GluA2 and was confirmed in luciferase experiments (Saba et al., 2012). In addition, miR-181a over-expression in hippocampal cultures reduced AMPA (α-amino-3-hydroxy-5-methyl-4-isoxazolepropionic acid) receptor surface clusters, spine volume, and mEPSC frequency. Finally, the association between miR-181a and drugs of abuse has been highlighted (Chandrasekar and Dreyer, 2011). In that study, lentiviral over-expression of miR-181a in the nucleus accumbens enhanced cocaine-induced conditioned place preference. Two studies performed on the miRNA expression in the dorsal horn of spinal cord in neuropathic pain models quantified a down-regulation of miR-181a (Li et al., 2013) or other members of the miR-181 family, namely miR-181b/c/d (von Schack et al., 2011). Thus, this miR-181 down-regulation could lead to an up-regulation of the excitatory receptor GluA2 in the nociceptive pathway.

Involvement of AMPA receptors in neuropathic pain mechanisms has already been suggested (for review, see Garry and Fleetwood-Walker, 2004). Unfortunately, the expression of GluA2 was not assessed in these two miRNA studies. Interestingly, another study on a different model, chronic pelvic pain, revealed a role of miR-181a through the regulation of a different target, the inhibitory receptor GABA_A__α__-__1_ (Sengupta et al., 2013). Pelvic pain was experimentally elicited by intra-vesicular injections of zymosan (a carbohydrate extracted from yeast and known to induce inflammation) into the bladder at postnatal day 14. This induced long-lasting neuroanatomical and neurophysiological changes in the nociceptive system still present at postnatal day 60. At that stage, zymosan-treated animals were characterized by visceral hypersensitivity, an up-regulation of miR-181a and a down-regulation of GABA_A__α__-__1_ expression in the lombo-sacral segment of the spinal cord. Bioinformatics analyses identified multiple sites for miR-181a/b in the 3′UTR of GABA_A__α__-__1_ and luciferase experiments proved the inhibitory effect of miR-181a on GABA_A__α__-__1_ translation. These results strongly suggest that this miR-181a-induced down-regulation of GABA_A_ receptors results in a loss of inhibition in the spinal cord sustaining long-lasting visceral hypersensitivity. They highlight the importance of target identification in miRNA studies.

*N*-methyl-D-aspartate receptors are another kind of glutamate receptor known to play a crucial role in synaptic transmission and plasticity. Altered NMDA-mediated signaling is associated with behavioral impairments in psychiatric disorders. Kocerha et al. (2009) demonstrated that miR-219 is down-regulated in the prefrontal cortex in response to pharmacological or genetic disturbances of NMDA signaling. In addition, they showed that miR-219 targets calcium/calmodulin-dependent protein kinase II (CaMKII) subunit γ, a downstream component of the NMDA signaling cascade. Thus, miR-219 down-regulation upon NMDA inhibition appears to be a compensatory mechanism that attenuates behavioral disorders associated with acute receptor antagonism.

Two miRNA screening studies suggest that miR-219 may be involved in pain mechanisms. The first evidence came from a model of traumatic spinal cord injury where miR-219 was significantly down-regulated 7 days after contusion (Liu et al., 2009). On the contrary, sciatic nerve ligation (CCI model) was demonstrated to induce an up-regulation of this miRNA (Genda et al., 2013). However, in both cases the investigation of targeted mRNA still needs to be conducted to shed light on the pathological role of miR-219 in pain pathways. In addition, the group of M. Sheng demonstrated that the expression of the NR2A subunit of NMDARs was regulated by miR-125b in hippocampal neurons (Edbauer et al., 2010). Indeed, endogenous miR-125b affected NMDAR composition and therefore induced functional changes in channel kinetics. As a consequence, miR-125b expression is likely to be critical for synaptic plasticity. Interestingly, a CCI pain model induced the down-regulation of miR-125b in the hippocampus (Arai et al., 2013), a structure that is part of the descending anti-nociceptive system.

miR-124 is one of the most abundant miRNAs in the brain and its role in the development of the mammalian brain is now well established. Thus, miR-124 is a neuronal differentiation promoter that also acts as an inhibitor of neuronal stem cells (Cheng et al., 2009; Yoo et al., 2009; Åkerblom et al., 2012). During *X. laevis* development, miR-124 plays a crucial role in timing the axon pathfinding of retinal ganglion cells (RGCs; Baudet et al., 2012). Thus, a key event in the correct navigation of RGC axons is a change in growth cone sensitivity to guidance cues. Baudet et al. (2012) demonstrated that miR-124 regulates the initiation of growth cone responsiveness to semaphorin Sema3A. In addition, miR-124 was shown to regulate neuronal process complexity by modulating the small GTPase RhoG (Franke et al., 2012). Indeed, RhoG is a critical modulator of the actin cytoskeleton. Consequently, RhoG inhibits axonal branching so miR-124-mediated inhibition of RhoG stimulates axonal and dendritic complexity. Yang et al. (2012) demonstrated that miR-124 is a direct regulator of the transcription factor Zif268, which is essential for activity-related modulation of synaptic transmission and cognition.

In the context of chronic pain, miR-124 down-regulation in DRG neurons was reported in inflammatory muscle pain (Bai et al., 2007), as well as in sciatic nerve after peripheral nerve crush (Wu et al., 2011). Moreover, intrathecal injection of miR-124 had an anti-nociceptive effect in both inflammatory and nerve injury-induced chronic pain models (Willemen et al., 2012). Finally, Niederberger’s group showed that intravenous injection of miR-124 alleviates the nociceptive response to the formalin test and identified the target involved as MeCP2 (Kynast et al., 2013). Thus, in addition to miR-132 (Klein et al., 2007), miR-124a also regulates MeCP2 translation. MeCP2 is an epigenetic modulator of BDNF, one of the major players in inflammatory pain mechanisms (Merighi et al., 2008).

One of the interesting characteristics of miRNAs that has been conserved through evolution is that a given miRNA has the potential to regulate multiple targets owing to the permissive hybridization to target mRNAs. This mechanism of action is now well accepted and it can have an important impact on gene expression regulation. Thus, one or few miRNAs could regulate the expression of multiple genes that participate in the same function or which constitute the different subunits of a macromolecular complex (Ferretti et al., 2008; Harraz et al., 2012).

In the context of chronic pain, we demonstrated such a multiple targeting by one miRNA. Indeed, we showed that a single miRNA, miR-103, simultaneously regulates the expression of the three subunits of the Cav1.2-comprising L-type calcium channel (Cav1.2-LTC) in an integrative regulation. Sensitization to pain involves the activation of various types of voltage-gated calcium channels. Some control synaptic transmission (Matthews and Dickenson, 2001; Bourinet et al., 2005; Altier et al., 2007) while others regulate intrinsic membrane properties. Among the latter, Cav1.2-LTC regulates gene expression underlying long-term plastic changes (Dolmetsch et al., 2001; Fossat et al., 2010). miR-103-mediated modulation of Cav1.2 function is bidirectional since knocking-down or over-expressing miR-103 respectively up- or down-regulate the level of Cav1.2-LTC translation. Functionally, we showed that miR-103 knockdown in naive rats results in hypersensitivity to pain. Moreover, miR-103 was down-regulated in neuropathic animals and miR-103 intrathecal applications successfully relieved pain, thus identifying miR-103 as a possible novel therapeutic target in neuropathic chronic pain (Favereaux et al., 2011).

### ACTIVITY-INDUCED MODULATION OF miRNA LEVELS

A set of evidence is growing to suggest that miRNAs can regulate neuronal activity by modulating the expression of ion channels, the morphology of dendritic spines and the molecular pathways downstream from receptor activation. However, the mechanisms that induce an activity-related modulation of miRNA-mediated inhibition of target genes remain poorly understood. Recent reports mostly in non-neuronal cells showed that it involves regulation of both the metabolism and function of miRNAs, and some of these mechanisms were also found to play a crucial role in neurons (for review, see Krol et al., 2010b). Below, we summarize the known mechanisms that modulate miRNA levels as a result of activity variations in neurons.

First, modulation of miRNA levels in response to activity could result from transcription regulation as for mRNAs. Indeed, Nomura et al. (2008) found that the transcription of miR-184, a brain-specific miRNA, is repressed by the binding of MeCP2. miR-184 is an imprinted gene exclusively expressed from the paternal allele. Interestingly, enhancement of neuronal activity by high-KCl treatment induced MeCP2 phosphorylation and the consequent release of phosphorylated MeCP2 from the miR-184 promoter region. Thus, the increase in miR-184 levels in response to neuronal activity involves at least an augmented transcription of miR-184.

CREB is a transcription factor that plays a crucial role in nervous system development and plasticity (reviewed in Lonze and Ginty, 2002). A genome-wide screen for CREB-binding sites identified hundreds of locations that are associated with non-coding RNAs and miRNAs, including miR-132 (Vo et al., 2005). DNase I footprinting assay confirmed the CREB binding at two consensus cAMP regulatory elements (CREs) in the close 5′ region of the miR-212/-132 locus. CREB is known to be activated by neurotrophins such as BDNF, and the authors demonstrated that miR-132 transcription was enhanced in response to BDNF. In addition, CREB inhibition partially blocked the BDNF-induced increase of miR-132, strongly suggesting that this mechanism involved the CREB pathway. Wayman et al. (2008) demonstrated that miR-132 is regulated by CREB in an activity-dependent manner. Hence, in hippocampal neuronal cultures, inhibition of GABA_A_ inhibitory tone with bicuculline increased synaptic activity and the levels of miR-132 precursor and mature forms. Interestingly, blockade of CREB-dependent transcription abolished the miR-132 up-regulation in response to synaptic activity.

Second, the function of miRNA can be modulated by regulating miRISC components. Hence, MOV10, the mammalian ortholog of the SDE3 helicase Armitage in *Drosophila* (Ashraf et al., 2006), is present at synapses and undergoes rapid proteolysis in response to NMDAR-mediated activity (Banerjee et al., 2009). As a consequence, it relieves miRNA-mediated translation inhibition of key proteins at the synapse like CaMKII or LIMK1 (Ashraf et al., 2006; Banerjee et al., 2009).

Another possible mechanism could be the activity-dependent regulation of dendritic P-bodies. Cougot et al. (2008) demonstrated that ribonucleoprotein particles (RNPs) with a P-body-like structure are present in dendrites and that they exhibit motorized movements upon synaptic activation to relocate to distant sites (Cougot et al., 2008). In addition, neuronal activation induced a loss of AGO2 from these P-body-like structures, suggesting that it may regulate local translation. Thus, inhibited messenger RNPs (mRNPs) may be stored in these particles at resting state and then be released in response to synaptic activity in order to be translated.

Besides regulation of miRISC components, recent evidence suggests that synergistic pathways may contribute to miRNA function and could therefore be activity-regulated. Hence, FMRP (fragile X mental retardation protein) is a protein with multiple RNA-binding domains that acts as a translational suppressor of particular mRNAs (Bassell and Warren, 2008). Interestingly, FMRP interacts with RISC proteins and miRNAs, although it is not necessary for miRNA-mediated inhibition (Caudy et al., 2002; Ishizuka et al., 2002; Jin et al., 2004). Edbauer et al. (2010) recently showed that both FMRP and miR-125b bound NR2A NMDAR subunit mRNA, thus leading to translation inhibition. Moreover, the inhibition of NR2A by FMRP is enhanced by miR-125b and FMRP depletion reversed miR-125b effects, strongly suggesting a synergistic effect.

Finally, obtaining a net change in miRNA levels without affecting miRNA transcription could be the result of miRNA decay alterations. Indeed, recent evidence suggests that miRNA turnover is fast in neuronal cells owing to rapid decay (Krol et al., 2010a). In addition, the authors demonstrated that the rate of miRNA decay is activity-dependent. Thus, blocking neuronal activity with either tetrodotoxin (TTX; inhibiting action potentials) or 6-nitro-sulfamoyl-benzo-quinoxaline-dione/3-carboxy- piperazin-propyl phosphonic acid (NBQX/CPP; inhibiting glutamate transmission) reduced miRNA decay. Some elements of the enzymatic machinery involved in miRNA degradation have recently been discovered (Ramachandran and Chen, 2008; Chatterjee and Grosshans, 2009). However, the factors responsible for the rapid and activity-dependent miRNA decay in neurons are still unknown and constitute a new challenge. The most recent breakthrough in our understanding of miRNA regulation in neurons came from an underestimated RNA class, circular RNAs (circRNAs). circRNAs were first discovered in plants (Sanger et al., 1976) and then in unicellular organisms (Grabowski et al., 1981), but the best-known circRNA is the sex-determining region Y RNA (Sry) which is highly expressed in testes (Capel et al., 1993). Recently, two groups identified a brain-expressed circRNA [(named CDR1as (cerebellar degeneration-related protein 1 antisense) or circular RNA sponge for miR-7 (ciRS-7)] that contains roughly 70 target sites for miR-7 (Hansen et al., 2013; Memczak et al., 2013). This circRNA acts as a natural miRNA sponge since it binds several miR-7 copies at a time, thus reducing miR-7 inhibition on mRNA targets. Interestingly, a very recent study identified miR-7 as a regulator of neuronal activity in chronic pain conditions. Hence, Sakai et al. (2013) showed that miR-7a was strongly down-regulated in the DRG of neuropathic pain models in animals. They demonstrated that miR-7a targets the β2-subunit of voltage-gated sodium channel, thus increasing the excitability of nociceptive neurons. In addition, miR-7a exogenous over-expression in DRG of injured animals alleviated neuropathic pain.

## CONCLUSION

How miRNAs regulate neuronal function in response to activity and whether deregulation of these mechanisms triggers pathological states are still poorly understood. For instance, are the miRNA pathways that control neuronal fate during development also involved in brain homeostasis? What are the mechanisms that could modulate miRNA expression in response to neuronal activity? Do the pathways involved in miRNA regulation depend on the kind of stimulus or on the miRNA species? What is the spatial resolution of miRNA control on local translation? What is the therapeutic potential of targeting activity-regulated miRNA?

The analysis of miRNA regulation in challenging pathological conditions such as chronic pain may help to highlight crucial miRNA mechanisms that are discreet in basal conditions. In the past few years, many studies have screened for miRNA expression in various animal models and in cohorts of patients with diverse neurodegenerative diseases. Although highlighting biomarker miRNAs with a possible value for therapeutic approaches, this kind of approach does not improve our understanding of miRNA targeting and function in pathological mechanisms. Indeed, it would be risky to evaluate targets of candidate miRNAs on algorithm-based prediction databases only, since the number of putative targets is very large and confirmation of targeting sometimes difficult to achieve. Therefore, it will be important to improve miRNA target prediction tools maybe by considering recent experimental data suggesting that “seed” region may not be the only signature of miRNA targeting (Helwak et al., 2013). Moreover, it would be interesting to compare miRNA and mRNA expression data as often as possible in the light of experimentally confirmed miRNA:mRNA interactions.

Nevertheless, the various experimental paradigms with their sometimes conflicting data could hinder progress toward a better understanding of miRNA function. Therefore, the use of unified experimental parameters could further clarify the role of miRNA in neuronal plasticity. The large body of pre-existing data provides solid evidence for the involvement of miRNA regulation in both physiological and pathological conditions. Thus, miRNA is an attractive potential therapeutic target.

## Conflict of Interest Statement

The authors declare that the research was conducted in the absence of any commercial or financial relationships that could be construed as a potential conflict of interest.
